# Integrin β1 subunit regulates cellular and secreted MUC5AC and MUC5B production in NCI–H292 human lung epithelial cells

**DOI:** 10.1016/j.bbrep.2021.101124

**Published:** 2021-09-01

**Authors:** Jun Iwashita, Jun Murata

**Affiliations:** Faculty of Bioresource Sciences, Akita Prefectural University, 241-438 Kaidobata-Nishi, Shimoshinjo-Nakano, Akita, Akita, 010-0195, Japan

**Keywords:** Asthma, Integrin β1, MUC5B, MUC5AC

## Abstract

The surface of the human respiratory tract is covered with a mucus layer containing mucin 5AC (MUC5AC) and mucin 5B (MUC5B) as the main components. This layer contributes to biological defense by eliminating irritants, but excessive MUC5AC secretion by the airway epithelial cells exacerbates asthma. Therefore, regulating mucin production is important for asthma treatment. In this study, the effects of integrin β1 subunit on MUC5AC and MUC5B production were examined in NCI–H292 human lung cancer epithelial cells. When integrin β1 was overexpressed, cellular and secreted MUC5AC levels were decreased, whereas cellular MUC5B production was increased. Conversely, integrin β1 depletion using siRNA increased cellular and secreted MUC5AC production, but decreased cellular MUC5B production. Further, the activity of extracellular signal-regulated kinase (ERK), which promotes MUC5AC production, was decreased by integrin β1 overexpression and increased by its depletion. These results suggest that integrin β1 suppresses MUC5AC production and promotes MUC5B production by downregulating ERK.

## Highlights

• We studied the regulation of MUC5AC and MUC5B production by integrin β1 subunit.
• Integrin β1 overexpression reduced MUC5AC, but increased MUC5B levels.
• Integrin β1 depletion increased MUC5AC production and ROS level, but decreased MUC5B production.
• Integrin β1 overexpression decreased ERK activity in NCI–H292 airway cells.
• Integrin β1 downregulates ERK to suppress MUC5AC & promote MUC5B production.

## 1 Introduction

The mucus layer secreted in human airways plays a key role in primary host defense against foreign matter [1]. A chronic disease, bronchial asthma, is characterized by excessive secretion of mucus in airway. The excessive mucus secretion causes airway narrowing and disease exacerbation of illness [2,3]. Hence, the suppression of excessive mucus secretion in airway leads to treatment of asthma.

The main component of airway mucus is mucin proteins. Mucin proteins are highly glycosylated and create stickiness of mucus. Mucins are produced and secreted in airway epithelial cells [4]. So far, at least 20 different mucin gene subfamilies were reported and 8 of them are expressed in human airway cells [5]. In human airway mucus, mucin 5AC (MUC5AC) and mucin 5B (MUC5B) are the major mucin subtypes. MUC5B is expressed throughout in normal human airway surface, with the exception of terminal bronchioles [6]. MUC5AC is hyperexpressed from goblet cells and increased airway mucus in asthma patients [[7], [8], [9], [10]].

So far, many reports on the regulation of MUC5AC production have been made as a method for asthma treatment [7]. In previous reports, inflammation of airways, some proinflammatory cytokines, cell adhesion molecules, Akt, and some flavonoids induce hypersecretion of airway mucus by inducing morphological and proliferative changes in airway epithelial cells [[11], [12], [13], [14], [15]]. Those stimulations which induce MUC5AC secretion are transmitted mainly through epidermal growth factor (EGF) receptors [16,17]. The signaling from EGF receptors activate the extracellular signal-regulated kinase (ERK) pathway and increase certain transcription factors, such as nuclear factor-κB and Sp1, which eventually increase MUC5AC production [1,18]. It has been reported that MUC5AC expression is upregulated by a TNF-α-converting enzyme/EGF receptor pathway, which is activated by reactive oxygen species (ROS) [19].

Airway epithelial cells are surrounded and supported by the extracellular matrix (ECM). ECM contains several proteins, such as laminins, fibronectins, and collagens [[20], [21], [22], [23], [24]]. Collagens, especially type IV collagen (Col4), are the most abundant proteins in ECM, and they provide structural support to resident cells, such as human airway epithelial cells, and play various roles in cell–cell communication. In our previous study, we demonstrated that certain ECM proteins were involved in the regulation of MUC5AC secretion. We previously found that MUC5AC production was reduced in NCI–H292 human lung cancer epithelial cells and human primary asthmatic lung cells cultured with Col4 [1,25]. However, the pathway that connects ECM proteins such as Col4, and the regulation of MUC5AC and MUC5B production has not yet been clarified. In this report, we analyzed the molecules which connect between ECM and MUC5AC production.

The typical receptors of ECM proteins are integrins. Integrins are heterodimeric receptors composed of noncovalently bound α and β subunits and are involved in signal transduction from ECM [26,27]. To date, at least 18 α subunits and 8 β subunits have been identified [26]. In addition, various combinations of integrin α and β subunits are possible, and the ECM component bound by integrins differs depending on the combination. There are two well-known combinations of integrins that recognize Col4, namely α1β1 and α2β1, but α2β1 has also been reported to recognize Col4 in NCI–H292 cells [28]. In our previous report, it was suggested that cell attachment and the level of integrin β1 subunit might regulate MUC5AC production [29]. However, the function of integrin β1 subunit in the regulation of MUC5AC production and secretion remains unclear.

In this study, the effects of integrin β1, which connects Col4 and cells, on MUC5AC and MUC5B production and secretion were analyzed in NCI–H292 cells.

## 2 Materials and methods

### 2.1 Cell culture

NCI–H292 cells were purchased from the American Type Culture Collection (Manassas, Virginia, USA). NCI–H292 cells were cultured in RPMI-1640 (Sigma-Aldrich, Tokyo, Japan) supplemented with 10% fetal bovine serum (FBS, Cansera International, Etobicoke, Canada), 100 units/ml penicillin (Gibco Oriental, Tokyo, Japan), and 100 μg/ml streptomycin (Gibco Oriental) in a 5% CO_2_ incubator (ShinMaywa, Tokyo, Japan). The adherent cells were subcultured every 3–4 days via treatment with a trypsin-EDTA solution (Gibco Oriental). Cells were seeded on two types of culture plates: a 96-well adhesive plate (MS-8096F, Sumilon, Tokyo, Japan) and a 96-well low-adhesive plate (MS8096R, Sumilon).

### 2.2 Gene transfer of integrin β1 and α2

NCI–H292 cells adjusted to a density of 1 × 10^4^ cells/well were seeded into 96-well plates in 100 μl of medium per well and precultured in an incubator at 37 °C for 24 h. Then, 95 μl of RPMI and 5 μl of cDNA were added to a 1.5-ml tube, the cDNA concentration was adjusted to 100 ng/well, and then 3 μl of X-treme gene transfection reagent (Roche Diagnostics, Hague Rd, IN, USA) was added and mixed. Subsequently, 10 μl of the mixed solution was added to each well. The plate was incubated at 37 °C for 6 h, followed by medium replacement and incubation at 37 °C for 48 h. Subsequently, the medium was removed and sampled. The treatment period and dose of cDNA were determined according to our previous paper or the manufacturer's instructions [29]. We adopted the optimum conditions of cDNA dose from among 50ng/well, 100ng/well and 200ng/well. Laemmli sample buffer (1 × , 0.0625 M Tris-HCl, 2% sodium dodecyl sulfate [SDS] solution, 5% SDS, 0.005% bromophenol blue, 5% 2-mercaptoethanol) was used for western blotting. The cDNA constructs of integrin β1 (β1 cDNA, 16042, pRK5 beta1, Addgene, Watertown, MA, USA), pRK5 vectors (control, CNTL), integrin α2 (α2 cDNA, 54128, mEmerald-Integrin-α2-N-18, Addgene) and mEmerald-N1 vectors (CNTL, 53976, mEmerald-N1, Addgene) were used for transfection.

### 2.3 siRNA transfection

siRNA transfections were performed with siRNA transfection Medium (Santa Cruz Biotechnology, Santa Cruz, CA, USA) and siRNA transfection Reagent (Santa Cruz Biotechnology). NCI–H292 cells were adjusted to a density of 1 × 10^4^ cells/well, and 100 μl of cells were seeded into each well of a 96-well plate followed by overnight culture. Transfection medium (11 μl/well) and integrin β1 siRNA (h) (1.2 μl/well, sc-35674, Santa Cruz Biotechnology), control siRNA-A (1.2 μl/well, sc-37007, Santa Cruz Biotechnology), ITGB1 siRNA (1.2 μl/well, AM16708, Thermo Fisher Scientific, Rockford, IL, USA), or Silencer Negative Control No. 1 siRNA (1.2 μl/well, AM4611, Thermo Fisher) were added to a 2.0-ml tube to prepare solution A. The siRNAs were diluted with RNAse-free water provided to the concentration of 10 μM. We adopted the optimum conditions of siRNA dose from among 0.6 μl/well, 1.2 μl/well and 1.8 μl/well. Subsequently, transfection medium (11 μl/well) and transfection reagent (0.6 μl/well) were added to a 2.0-ml tube to prepare solution B. Solution A was mixed in equal amounts with solution B and allowed to stand for 30 min, after which 82 μl of the transfection medium was added to tube. The culture medium was removed from the 96-well plate, 100 μl/well of mixed solution was added to each well, and the mixture was allowed to stand for 6 h. Next, the siRNA solution was removed, the culture medium was added to each well at 100 μl/well, and the cells were cultured for 48 h. Subsequently, the culture medium was removed, and 1 × Laemmli sample buffer (20 μl/well) and 0.1% Dot Blot SDS in Tris-buffered saline (TBS, 150 mM NaCl, 10 mM Tris pH 7.5) containing 0.2% Tween 20 (TBS-T) were added at 100 μl/well for sample preparation. The treatment period and dose of cDNA were determined according to our previous paper or the manufacturer's instructions [29].

### 2.4 MUC5AC and MUC5B protein measurement using the dot blot method

NCI–H292 cells were washed once with culture medium and suspended in the medium using a syringe with a 26G needle to obtain a single-cell suspension. Diluted cells (1 × 10^4^ cells in 100 μl of medium) were added to the wells and incubated for 3–30 h at 37 °C. After removing and reserving the culture medium to measure secreted mucin levels, the cells were harvested via lysis in TBS containing 0.1% SDS. In total, 10 μl of the solution was blotted onto an Immobilon membrane (Millipore, Temecula, CA, USA) using a Dot Blot Hybridization Manifold (48-well, SCIE-PLAS, Cambridge, UK). When measuring soluble MUC5AC and MUC5B levels in the culture medium, the removed culture medium was used as the sample and applied onto an Immobilon membrane. The membrane was treated with Western blot blocking buffer (T7131A, Takara, Tokyo, Japan) in TBS-T for 12 h at 4 °C and then incubated with mouse antihuman MUC5AC antibody (MS145-P1, 1:2000 in Western blot blocking buffer, Neomarkers, Fremont, CA, USA) or mouse antiMUC5B antibody (ab77995, 1:2000 in 4% skim milk, Abcam, Tokyo, Japan) for 1 h. The membrane was washed five times for 5 min each with TBS-T and then incubated with rabbit antimouse IgG (H + L) (1:2000 in 4% skim milk, NA931V, GE Healthcare, Buckinghamshire, UK) for 1 h. After washing the membrane five times, enzyme reactions were detected using Luminata Forte Western HRP Substrate (WBLUF0500, Millipore) and a ChemiDoc image analyzer (Bio-Rad, Tokyo, Japan).

### 2.5 Cell proliferation assay

Cell proliferation was assessed using Cell Counting Kit-8 (Dojindo, Kumamoto, Japan). After adding the kit reagent (0.01 ml/0.1 ml) to each well, the plate was incubated for 2 h at 37 °C. Cell growth was assessed by measuring absorbance at 450 nm using a Model 550 microplate reader (Bio-Rad).

### 2.6 Measurement of cellular ROS

ROS production in NCI–H292 cells was measured following gene transfer or siRNA transfection of integrin β1. For cellular ROS quantification, Amplex red hydrogen peroxide/peroxidase assay kit (Invitrogen A22188) was used and measurement were performed according to the manufacturer's instructions. The converted ROS was assessed by measuring absorbance at 550 nm using a Model 550 microplate reader (Bio-Rad).

### 2.7 Immunohistochemistry

For immunofluorescence analyses, transfected cells (3.1 × 10^4^ cells/200 μl) were seeded in chamber slides (177445, Lab-Tek™ Nalge Nunc International, Rochester, NY, USA). After 48 h of culture, the culture medium was removed, and the cells were washed once with phosphate-buffered saline (PBS). Subsequently, the cells were fixed in methanol for 10 min at −20 °C and treated with acetone for 5 min at −20 °C. The cells were blocked with 1% bovine serum albumin in PBS for 30 min at room temperature and washed three times with PBS. The fixed and rinsed cells were incubated with mouse monoclonal antiMUC5AC antibody or mouse antiMUC5B antibody as the primary antibody (1:1500 dilution in PBS) for 1 h at room temperature. Then, the cells were washed three times with PBS and incubated with antimouse IgG-FITC (#238, MBL, Nagoya, Japan) as the secondary antibody (1:1500 dilution in PBS) for 1 h at room temperature. After being washed in PBS, the slides were immediately viewed using a fluorescence microscope (BZ-9000, Keyence, Tokyo, Japan).

### 2.8 Inhibition of ERK pathway

U0126 (Wako, Tokyo, Japan), an inhibitor of the MEK/ERK pathway, was dissolved in 10 mM in dimethylsulfoxide (DMSO). U0126 was added to the cell culture medium after the removal of siRNA solution to a final concentration of 10 μM and cultured for 48 h at 37 °C. The same concentration of DMSO was added to the controls.

### 2.9 Immunoblot detection of integrin β1, β-actin, phosphorylated ERK, and total ERK

Cellular proteins were electrophoresed on a 10% SDS-PAGE gel using a CM-1005 apparatus (Cima Biotech, Tokyo, Japan) and then blotted onto a nitrocellulose membrane (Hybond ECL, GE Healthcare) using an M3001 transfer apparatus (Cima Biotech). The membrane was treated with Western blot blocking buffer in TBS-T for 12 h at 4 °C and then incubated with rabbit anti-integrin β1 polyclonal antibody (4706S; Cell Signaling Technology Japan, Tokyo, Japan), rabbit antiphosphorylated ERK1/2 antibody (GTX24819, Funakoshi, Tokyo, Japan), or rabbit antiERK 1/2 antibody (V1141, Promega, Madison, WI, USA) at a 1:2000 dilution in Western blot blocking buffer for 1 h. The membrane was washed five times for 5 min each with TBS-T and then incubated with antirabbit IgG antibody conjugated with horseradish peroxidase (W4011, Promega) at a 1:2000 dilution for 1 h. After washing the membrane five times, enzyme reaction was detected using Luminata Forte Western HRP Substrate and a ChemiDoc image analyzer. Cellular β-actin was detected as a control using rabbit anti–β-actin antibody (A5316, Sigma-Aldrich, St. Louis, MO, USA) at a 1:2000 dilution and antirabbit IgG antibody conjugated with horseradish peroxidase at a 1:2000 in Western blot blocking buffer. Subsequently, the blotted membrane was incubated with Restore Western Blot Stripping Buffer (21059, Thermo Fisher Scientific) for 15 min at room temperature in shaking conditions. The membrane was washed five times for 5 min each with TBS-T and then treated with Western blot blocking buffer for 12 h at 4 °C for reblocking.

### 2.10 Statistical analysis

Statistical analyses of the differences between the experimental groups were performed using analysis of variance and two-tailed unpaired Student's t-tests. A *p-*value of <0.05 was considered significant. All experiments were performed at least three times. The representative results are presented hereafter.

## 3 Results

### 3.1 Effects of integrin β1 or α2 overexpression on cellular and secreted MUC5AC and MUC5B levels

**Fig. 1:**
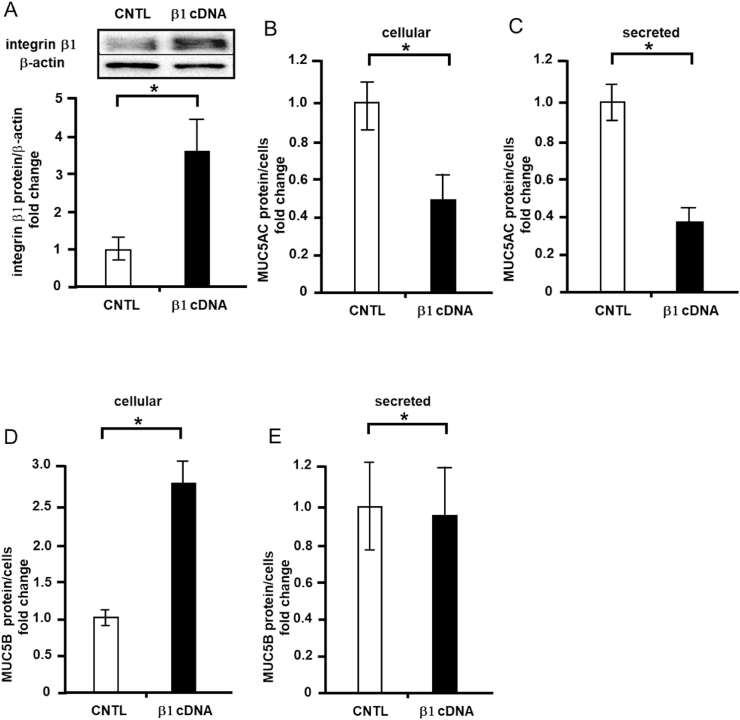
Effects of integrin β1 overexpression on cellular and secreted mucin 5AC (MUC5AC) and mucin 5B (MUC5B) levels in NCI–H292 cells. A, NCI–H292 cells were transfected with integrin β1 (β1 cDNA) or control vectors (CNTL) and cultured on a 96-well plate. Integrin β1 and β-actin protein levels were detected in cells via immunoblotting with specific antibodies, and their levels were measured using ChemiDoc. B, MUC5AC levels in transfected cells were detected using the dot blot method and measured (cellular). C, MUC5AC levels were detected in the culture medium of transfected cells via the dot blot method and measured (secreted). Fold changes were based on control cells (mean ± standard deviation [SD], n = 5). D, MUC5B levels in the transfected cells were detected using the dot blot method and measured (cellular). E, MUC5B levels in the culture medium of the transfected cells were detected using the dot blot method and measured (secreted). Fold changes were based on control cells (mean ± SD, n = 5). Fold changes were normalized for cell numbers. Student's t-test was used to obtain *p*-values. Asterisks indicate statistical significance, *p < 0.05. Representative results for three independent experiments are presented.

**Fig. 2:**
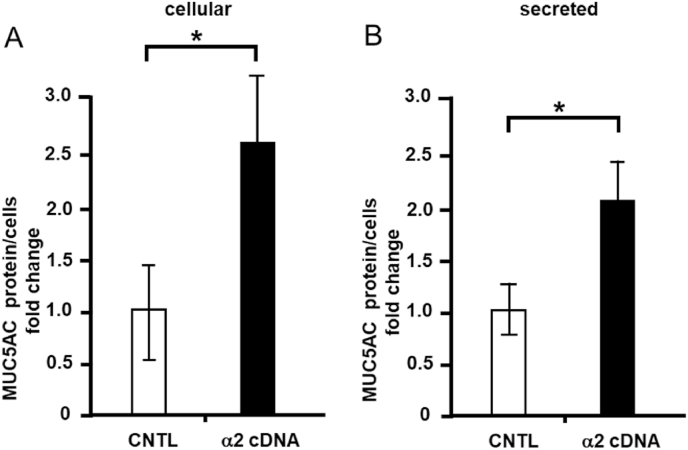
Effects of integrin α2 overexpression on cellular and secreted mucin 5AC (MUC5AC) levels in NCI–H292 cells. A, NCI–H292 cells were transfected with integrin α2 (α2 cDNA) or control vectors (CNTL) and cultured on a 96-well plate. A, MUC5AC levels in transfected cells were detected using the dot blot method and measured (cellular). B, MUC5AC levels were detected in the culture medium of transfected cells via the dot blot method and measured (secreted). Fold changes were based on control cells (mean ± standard deviation [SD], n = 5). Fold changes were based on control cells (mean ± SD, n = 5). Fold changes were normalized for cell numbers. Student's t-test was used to obtain *p*-values. Asterisks indicate statistical significance, *p < 0.05. Representative results for three independent experiments are presented.

First, to investigate the effects of integrin β1 on MUC5AC production, we transfected an integrin β1 gene construct into NCI–H292 cells via lipofection, and the protein expression of integrin β1 was measured via western blotting. Integrin β1 expression was increased by 3.5-fold following gene construct transfection compared to the findings for the control (empty vector transfection, Fig. 1A). In addition, integrin β1 overexpression decreased MUC5AC secretion by approximately 50% (Fig. 1B), but decreased MUC5AC secretion by approximately 60% (Fig. 1C) compared to the control level. Second, cellular and secreted MUC5B protein levels were measured. Following integrin β1 overexpression, cellular MUC5B secretion was increased by approximately 3-fold compared to the control level (Fig. 1D), whereas MUC5B levels were unaltered (Fig. 1E). In contrast, integrin α2 overexpression increased cellular MUC5AC protein expression by approximately 2.6-fold (Fig. 2A) and secreted MUC5AC expression by approximately 2.1-fold compared with the control (Fig. 2B).

### 3.2 Effects of integrin β1 depletion on cellular and secreted MUC5AC levels

**Fig. 3:**
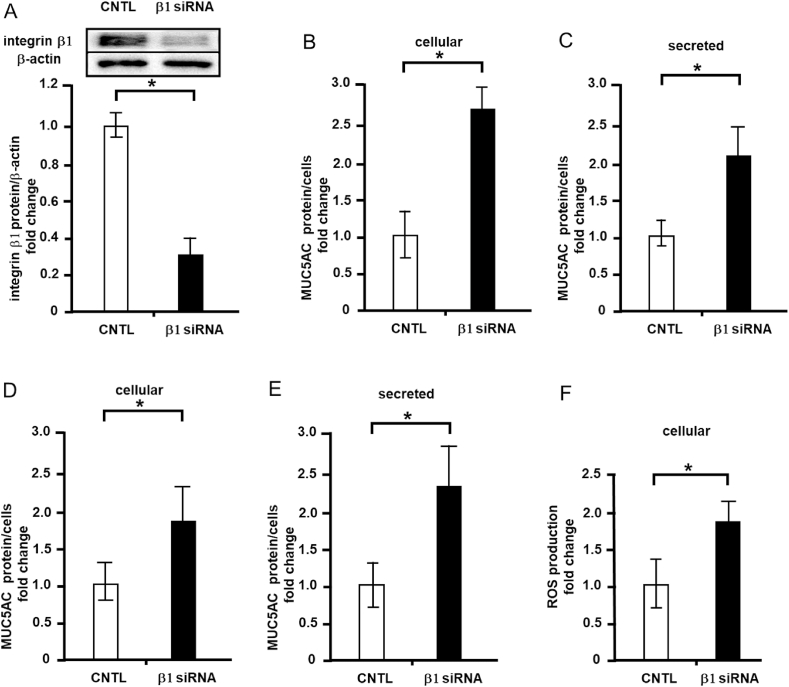
Effects of integrin β1 depletion on cellular and secreted mucin 5AC (MUC5AC) and ROS levels in NCI–H292 cells. A, NCI–H292 cells were transfected with integrin β1 siRNA (β1 siRNA, Santa Cruz Biotechnology) or control siRNA (CNTL), and cultured on a 96-well plate. Integrin β1 and β-actin protein levels were detected via immunoblotting and measured using ChemiDoc. B, MUC5AC levels in the integrin β1 siRNA (β1 siRNA, Santa Cruz Biotechnology) or control siRNA (CNTL), transfected cells were detected using the dot blot method and measured (cellular). C, MUC5AC levels in the culture medium of the in the integrin β1 siRNA (β1 siRNA, Santa Cruz Biotechnology) or control siRNA (CNTL), transfected cells were detected via the dot blot method and measured (secreted). D, MUC5AC levels in the integrin β1 siRNA (β1 siRNA, Thermo Fisher) or control siRNA (CNTL), transfected cells were detected using the dot blot method and measured (cellular). E, MUC5AC levels in the culture medium of the integrin β1 siRNA (β1 siRNA, Thermo Fisher) or control siRNA (CNTL), transfected cells were detected via the dot blot method and measured (secreted). F, ROS levels in the integrin β1 siRNA (β1 siRNA, Santa Cruz Biotechnology) or control siRNA (CNTL), transfected cells were detected by hydrogen peroxide/peroxidase assay (cellular). Fold changes were based on control cells (mean ± standard deviation, n = 5). Fold changes were normalized for cell numbers. Student's t-test was used to obtain *p*-values. Asterisks indicate statistical significance, *p < 0.05. Representative results for three independent experiments are presented.

First, siRNA for integrin β1 (Santa Cruz Biotechnology) was transfected into NCI–H292 cells, and integrin β1 protein levels were measured via western blotting. Intrinsic integrin β1 expression was decreased by 70% relative to the control level (Fig. 3A). In contrast, integrin β1 depletion increased cellular MUC5AC protein expression by 2.5-fold (Fig. 3B) and secreted MUC5AC expression by approximately 2-fold compared with the control (Fig. 3C). These depletion experiments were performed with another set of siRNAs for integrin β1 (Thermo Fisher) and similar results were obtained. The depletion of integrin β1 increased cellular (Fig. 3D) and secreted MUC5AC levels (Fig. 3E) compared to the control level.

### 3.3 Effects of integrin β1 depletion on cellular ROS level

It was reported that ROS intervened pathway induces MUC5AC production. Therefore, we transfected siRNA for integrin β1 (Santa Cruz Biotechnology) into NCI–H292 cells, and measured cellular ROS levels. The integrin β1 depletion increased cellular ROS level by 1.8-fold (Fig. 3F) relative to the control level.

### 3.4 Effects of integrin β1 depletion on cellular and secreted MUC5B levels

**Fig. 4:**
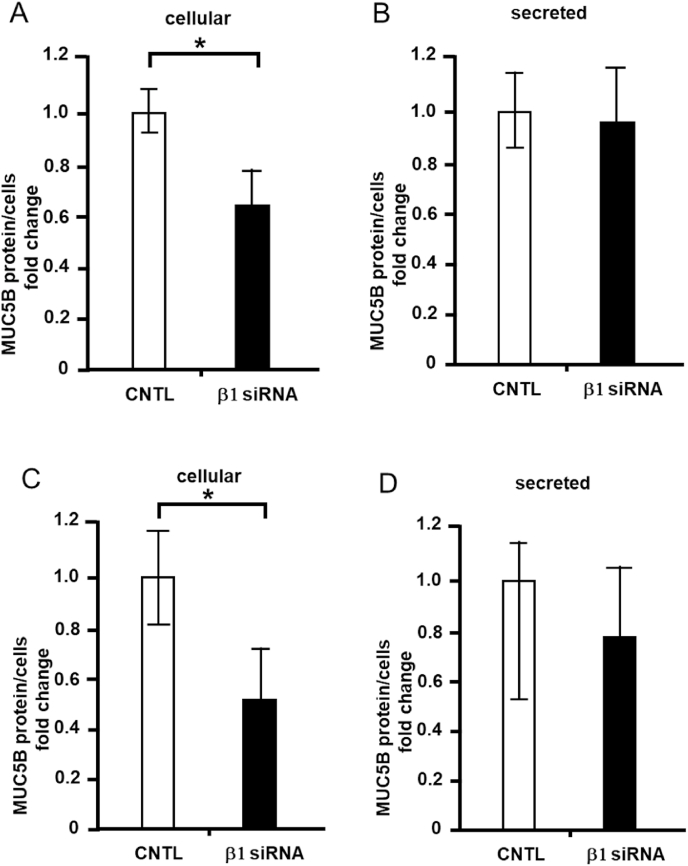
Effects of integrin β1 depletion on cellular and secreted mucin 5B (MUC5B) levels in NCI–H292 cells. A, MUC5B levels in the integrin β1 siRNA (β1 siRNA, Santa Cruz Biotechnology) or control siRNA (CNTL) transfected cells were detected via the dot blot method and measured (cellular). B, MUC5B levels in the culture medium of the integrin β1 siRNA (β1 siRNA, Santa Cruz Biotechnology) or control siRNA (CNTL), transfected cells were detected via the dot blot method and measured (secreted). C, MUC5B levels in the integrin β1 siRNA (β1 siRNA, Thermo Fisher) or control siRNA (CNTL), transfected cells were detected via the dot blot method and measured (cellular). D, MUC5B levels in the culture medium of the integrin β1 siRNA (β1 siRNA, Thermo Fisher) or control siRNA (CNTL), transfected cells were detected via the dot blot method and measured (secreted). Fold changes were based on control cells (mean ± standard deviation, n = 5). Fold changes were normalized for cell numbers. Student's t-test was used to obtain *p*-values. Asterisks indicate statistical significance, *p < 0.05. Representative results for three independent experiments are presented.

The NCI–H292 cells were depleted integrin β1 with siRNA (Santa Cruz Biotechnology) and cellular and secreted MUC5B protein expression was measured. The depletion of integrin β1 reduced cellular MUC5B levels by 40% compared to the control (Fig. 4A), but did not alter secreted MUC5B levels (Fig. 4B). These experiments were also performed with another set of siRNAs for integrin β1 (Thermo Fisher). The depletion of integrin β1 reduced cellular MUC5B levels compared to the control (Fig. 4C), but did not alter secreted MUC5B levels (Fig. 4D).

### 3.5 Immunostaining of cellular MUC5AC and MUC5B

**Fig. 5:**
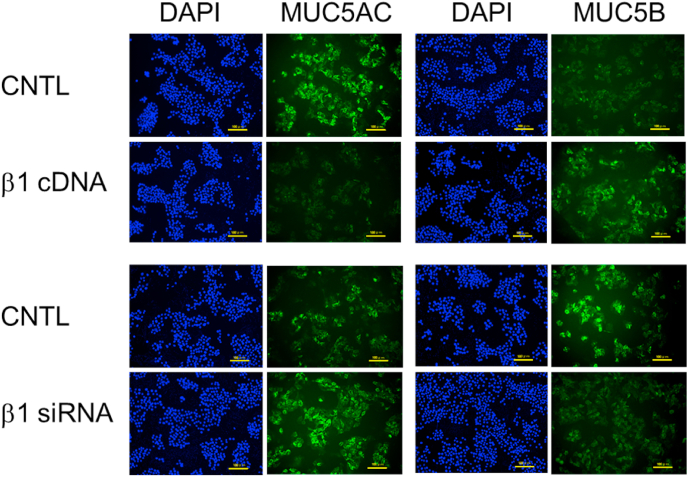
Effects of integrin β1 overexpression or depletion on cellular mucin 5AC (MUC5AC) and mucin 5B (MUC5B) levels in NCI–H292 cells. NCI–H292 cells (3.1 × 10^4^ cells/cm^2^) were transfected with integrin β1 vector (β1 cDNA), control vector (CNTL), integrin β1 siRNA (β1 siRNA), or control siRNA (CNTL), and cultured on Lab-Tek Chamber Slides for 48 h MUC5AC and MUC5B protein expression was detected via immunohistochemical analysis (green). Cell nuclei were stained with 4′,6-diamidino-2-phenylindole (DAPI, blue). Yellow bars indicate 100 μm. (For interpretation of the references to colour in this figure legend, the reader is referred to the Web version of this article.)

Our results suggest that integrin β1 overexpression downregulates cellular MUC5AC expression, whereas integrin β1 depletion upregulates cellular MUC5AC expression. Further, integrin β1 overexpression and depletion had opposite effects on cellular MUC5B expression. Therefore, we investigated cellular MUC5AC and MUC5B levels by immunostaining with antiMUC5AC and antiMUC5B antibodies. The microscopy results corroborated the results of the dot blot experiment. Cellular MUC5AC expression was decreased and cellular MUC5B expression was increased by integrin β1 overexpression (Fig. 5). In contrast, cellular MUC5AC expression was increased and cellular MUC5B expression was decreased by integrin β1 depletion (Fig. 5).

### 3.6 Effects of integrin β1 overexpression and depletion on ERK activity in NCI–H292 cells

**Fig. 6:**
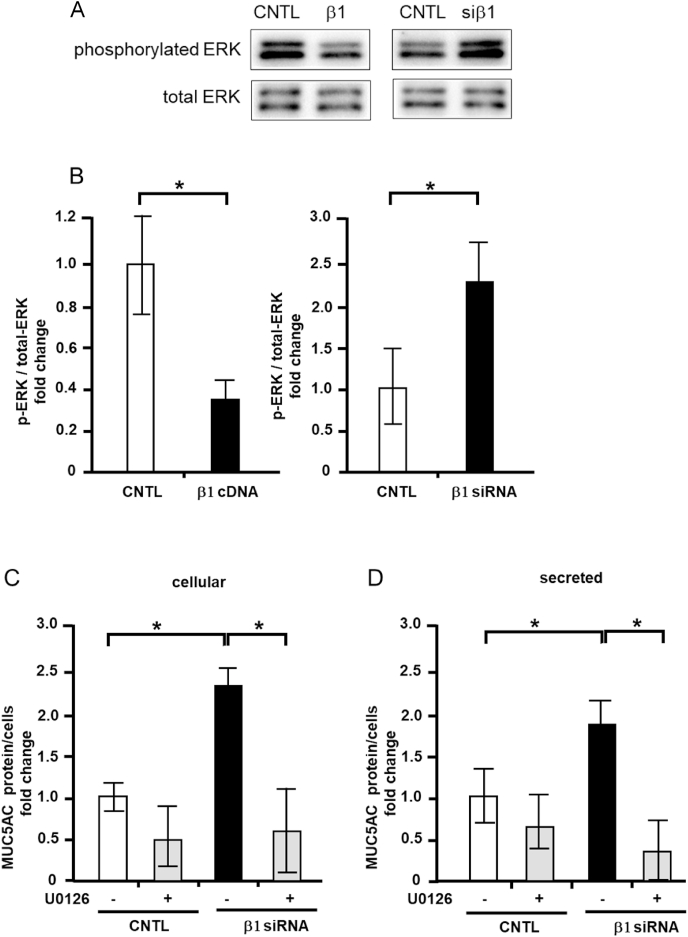
Activities of extracellular signal-regulated kinase (ERK) in NCI–H292 cells transfected with integrin β1 cDNA (β1 cDNA) or siRNA (β1 siRNA). A, NCI–H292 cells (1 × 10^4^ cells/well) were transfected with integrin β1 cDNA or siRNA, cultured, and sampled in a 96-well plate. Phosphorylated ERK and total ERK protein levels were detected. Representative results for three independent experiments are presented. B, A densitometry analysis of phosphorylated ERK/total ERK in NCI–H292 cells which were transfected with integrin β1 cDNA or siRNA. C, D, NCI–H292 cells were transfected with integrin β1 siRNA (β1 siRNA, Santa Cruz Biotechnology), control siRNA (CNTL) and cultured with (+) or without (−) 10 μM of U0126 on a 96-well plate. The cellular (cellular, C) and secreted (secreted, D) MUC5AC levels were detected via the dot blot method and measured.

**Fig. 7:**
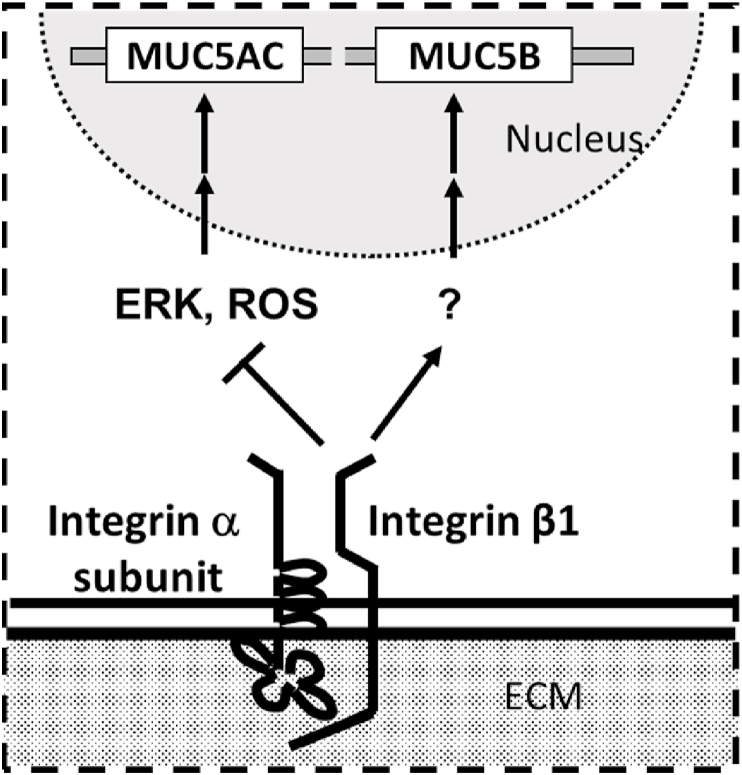
A hypothetical model of integrin β1 reduces MUC5AC expression via ERK activation and ROS formation. The arrow-headed lines and bar-headed lines indicate activation and inhibition, respectively.

Prior research has suggested that ERK promotes MUC5AC production via the integrin pathway in NCI–H292 cells [11]. We therefore measured the levels of phosphorylated (activated) ERK via western blotting. Integrin β1 overexpression suppressed ERK phosphorylation and activation, whereas integrin β1 depletion induced ERK phosphorylation and activation (Fig. 6A and B). Next, we inhibited ERK pathway in integrin β1 depleted cells, with an inhibitor, U0126. The cells were depleted integrin β1 with siRNA (Santa Cruz Biotechnology) and were treated with or without U0126 and measured cellular and secreted MUC5AC level. The elevated cellular and secreted MUC5AC level was reduced to control level by U0126 treatment (Fig. 6C and D). These results suggest that integrin β1 regulates MUC5AC expression via ERK intervening pathway (Fig. 7).

## 4 Discussion

Controlling MUC5AC production is an important aspect of asthma treatment; therefore, elucidating the regulatory mechanisms underlying MUC5AC secretion will lead to the development of novel therapies. We previously reported that MUC5AC production was reduced by Col4 signaling; however, to date, no reports have described the involvement of integrin α and β subunits, which connect Col4 to cells, in cellular MUC5AC production and MUC5AC secretion. Therefore, in this study, we investigated the effects of integrin β1 on the cellular production and secretion of MUC5AC and MUC5B in NCI–H292 cells. The study results suggest that integrin β1 suppresses MUC5AC production and secretion but promotes cellular MUC5B production in NCI–H292 cells. In addition, the findings indicated that the production and secretion of MUC5AC and MUC5B are regulated independently by integrin β1. MUC5B is the main component involved in biodefense in a healthy respiratory tract [6]. Thus, simultaneously increasing MUC5B and decreasing MUC5AC by upregulating integrin β1 may improve health.

Interestingly, integrin α2 overexpression increased cellular and secreted MUC5AC protein expression. These results suggest that integrin α2 increases MUC5AC production in contrast to the function of integrin β1. We plan to further analyze the function of integrin α2 subunit on MUC5AC and MUC5B production.

To the best of our knowledge, only one study has reported that MUC5AC is associated with integrin in the airway. MUC5AC interacts with integrin β4 and enhances the migration of lung cancer cells through focal adhesion kinase signaling [30]. This suggests that MUC5AC directly interacts with integrin; however, this report does not analyze the relationship between integrins and MUC5AC production and secretion. Thus, we believe that the present study is the first to report that MUC5AC production is controlled by a direct signal from the integrin subunits.

Integrin heterodimers expressed in the cell membrane regulates ERK activity [1,31]. Integrin β1 subunit overexpression downregulated ERK activity and reduced MUC5AC production. Moreover, depletion of integrin β1 subunit increased cellular and secreted MUC5AC, but this increase was suppressed by the inhibition of ERK activity. The suppressive effect of integrin β1 on MUC5AC production might be regulated through downregulation of ERK activity. We previously reported that Akt downregulates MUC5AC production in NCI–H292 cells [12]. Because it is possible that Akt regulates MUC5AC and MUC5B production and secretion, we plan to investigate Akt activity, which was modified by integrin β1 expression, in cells in the future.

Integrins function by forming heterodimers, but in this study, alterations in MUC5AC production were observed even when only integrin β1 was overexpressed. This result suggests the existence of free integrin α2 in cells, which is available for binding to the increased integrin β1 content, thereby leading to the suppression of MUC5AC production and secretion. We plan to analyze the effects of integrin α2 on MUC5AC and MUC5B production and secretion in the future.

In recent years, there are reports on the relationship between SARS CoV2 infection and integrins. The cellular β1, αvβ3 and αvβ6 integrins support adhesion of SARS CoV2 to human and primary mouse epithelial cells [32,33]. Therefore, the upregulation of MUC5B expression by integrin β1 subunit might be useful to the inhibition of SARS CoV2 infection.

ROS formation is reported to increase MUC5AC production in several cell lines such as NCI–H292 [19]. Our findings that the depletion of integrin β1 increases ROS level and integrin β1 downregulates MUC5AC production, suggest that integrin β1 inhibits ROS formation in mitochondria and represses MUC5AC expression. Therefore, the antioxidant therapy might be useful in the control of MUC5AC production in asthma treatment.

Our results suggest that the downregulation of MUC5AC production and secretion can be induced by integrin β1 subunit overexpression. This finding could facilitate the development of effective treatments to reduce airway mucus secretion in patients with asthma.

## 5 Conclusion

Our results suggest that integrin β1 subunit reduces MUC5AC cellular production and secretion but increases cellular MUC5B in NCI–H292 cell line. This decrease of MUC5AC production by integrin β1 subunit might be regulated by repression of ERK activity and ROS level.

## Author contributions

Jun Murata discussed the data.

## Funding information

This work was supported by the MEXT/JSPS KAKENHI Grant Number JP19K05882.

## Declaration of competing interest

The authors declare no conflicts of interest involving this article.
